# Molecular Understanding of ACE-2 and HLA-Conferred Differential Susceptibility to COVID-19: Host-Directed Insights Opening New Windows in COVID-19 Therapeutics

**DOI:** 10.3390/jcm12072645

**Published:** 2023-04-01

**Authors:** Ihtisham Ul Haq, Katarzyna Krukiewicz, Hamnah Tayyab, Imran Khan, Mehtab Khan, Galal Yahya, Simona Cavalu

**Affiliations:** 1 Department of Physical Chemistry and Technology of Polymers, Silesian University of Technology, 44-100 Gliwice, Poland; 2 Department of Biosciences, COMSATS University Islamabad (CUI), Islamabad 44000, Pakistan; 3 Joint Doctoral School, Silesian University of Technology, 44-100 Gliwice, Poland; 4 Centre for Organic and Nanohybrid Electronics, Silesian University of Technology, 44-100 Gliwice, Poland; 5 Department of Internal Medicine, King Edward Medical College, Lahore 54000, Pakistan; 6 Department of Molecular Signal Processing, Leibniz Institute of Plant Biochemistry, 06120 Halle, Germany; 7 Department of Biology, University of Moncton, Moncton, NB E1A 3E9, Canada; 8 Department of Microbiology and Immunology, Faculty of Pharmacy, Zagazig University, Zagazig 44519, Egypt; 9 Department of Molecular Genetics, Faculty of Biology, Technical University of Kaiserslautern, 67663 Kaiserslautern, Germany; 10 Faculty of Medicine and Pharmacy, University of Oradea, 410087 Oradea, Romania

**Keywords:** COVID-19, pandemic, genetic susceptibility, ACE-2, HLA, disease severity, antiviral immunity

## Abstract

The genetic variants of HLAs (human leukocyte antigens) play a crucial role in the virus–host interaction and pathology of COVID-19. The genetic variants of HLAs not only influence T cell immune responses but also B cell immune responses by presenting a variety of peptide fragments of invading pathogens. Peptide cocktail vaccines produced by using various conserved HLA-A2 epitopes provoke substantial specific CD8+ T cell responses in experimental animals. The HLA profiles vary among individuals and trigger different T cell-mediated immune responses in COVID-19 infections. Those with HLA-C*01 and HLA-B*44 are highly susceptible to the disease. However, HLA-A*02:01, HLA-DR*03:01, and HLA-Cw*15:02 alleles show resistance to SARS infection. Understanding the genetic association of HLA with COVID-19 susceptibility and severity is important because it can help in studying the transmission of COVID-19 and its physiopathogenesis. The HLA-C*01 and B*44 allele pathways can be studied to gain insight into disease transmission and physiopathogenesis. Therefore, integrating HLA testing is suggested in the ongoing pandemic, which will help in the rapid identification of highly susceptible populations worldwide and possibly acclimate vaccine development. Therefore, understanding the correlation between HLA and SARS-CoV-2 is critical in opening new insights into COVID-19 therapeutics, based on previous studies conducted.

## 1. Introduction

In December 2019, numerous patients in Wuhan, China presented with pneumonia and the causative agent was found to be a novel coronavirus [1,2,3]. Later investigations based on real-time PCR (polymerase chain reaction) led to the identification of a novel coronavirus [4,5]. Officially, the new coronavirus was discovered on the 7th of January 2020 and given the name nCoV-19 (novel coronavirus 2019) by the WHO (World Health Organization) [6]. The viral nomenclature, ICTV (International Committee on Taxonomy of Viruses) later replaced the nCoV-19 with severe acute respiratory syndrome coronavirus-2 (SARS-CoV-2) as it was genetically parallel to the previous SARS coronavirus. The disease caused by SARS-CoV-2 was named COVID-19 (coronavirus disease 2019) officially by the WHO [7]. Owing to the high virulence, pathogenicity, and contagiousness of SARS-CoV-2, in the second week of March 2020, the WHO declared COVID-19 as a pandemic [8]. The COVID-19 outbreak caused by SARS-CoV-2 received a great deal of interest across the world [9]. As of February 21st, 2023, more than 757, 264, 511 cases had occurred from the COVID-19 disease [10].

Since the outbreak of COVID-19, several approaches were proposed for finding potential therapeutics [11,12,13,14,15,16], until the development of effective anti-COVID-19 vaccines [17].

However, crucial questions about individual genetic variability in immune responses, disease susceptibility, and severity, as well as the clinical picture of the pandemic, remain unanswered [18]. In the past, the genetic-based approach of the HLA system, including the HLA-class I and II systems, has been used to trace the etiological agent of newly emerging infectious diseases and its interface with the variations in clinical outcomes of SARS-CoV disease [19]. Given its role in SARS-CoV and other viral diseases, such as MERS CoV (HLA-DRB1*11:01 and DQB1*02:02) [20], influenza (HLA-DRB1*0401 and HLA-DRB1*0402) [21], dengue (HLA-DRB1 * 0901 and HLA-A*24) [18], and hepatitis B (HLA- DPB1*09:01, DPB1*04:01, and DQB1*06:01) [22], the HLA system could also be beneficial in treating COVID-19 infections. The HLA genetic variant encodes hundreds of protein-coding genes that regulate the fundamental molecular and cellular processes, specifically the immune responses [23].

One study found that certain HLA alleles, such as HLA-B46:01 and HLA-B15:03, were associated with a lower risk of severe COVID-19, while other alleles, such as HLA-B07:02, were associated with an increased risk [24]. Another study found that HLA-A02:01, HLA-B07:02, and HLA-C07:29 were associated with a decreased risk of severe COVID-19, while HLA-B35:01 and HLA-C04:01 were associated with an increased risk [25]. These findings suggest that HLA variants may influence the immune response to SARS-CoV-2 infection and contribute to the variability in disease severity observed among individuals. It is currently unclear whether HLA variants arise in response to specific viral stimuli or occur in the context of most viral infections. However, some studies have shown that certain HLA alleles are associated with the protection or susceptibility to other viral infections. For example, HLA-B57:01 has been associated with slower HIV disease progression, while HLA-B15:05 has been associated with susceptibility to hepatitis B virus infection [26]. These findings suggest that HLA variants may be shaped by viral selection pressures.

**Figure 1:**
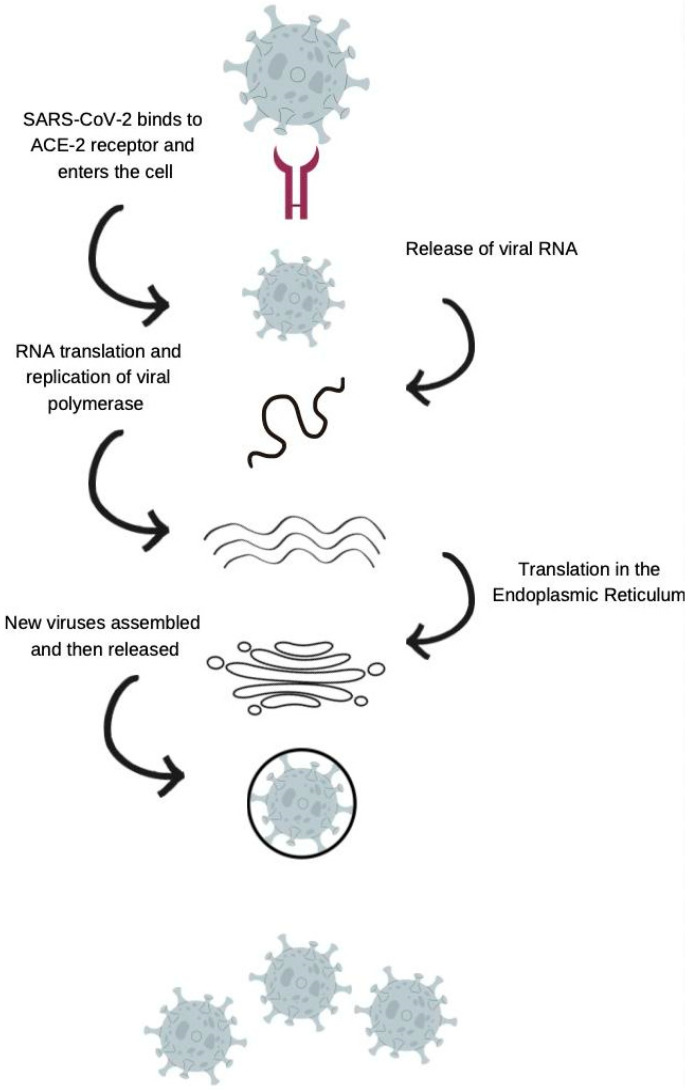
The attachment of SARS-CoV-2 virus on human ACE-2.

Furthermore, the ACE-2 receptor has played a significant role in the immune and inflammatory factors associated with COVID-19 pathogenesis. ACE-2, a homolog of ACE, is produced by several human organs and tissues and has a broad spectrum of biological functions [27]. A spike glycoprotein on the coronavirus’s viral envelope adheres to ACE-2 on the membrane of host cells that express ACE-2. After binding, the virus and host cell fuse their membranes, which activates infection by releasing viral RNA into the cytoplasm (Figure 1). Studies have shown that COVID-19 infections can reduce ACE-2 expression on cells, which might cause significant organ damage by disrupting the normal equilibrium between ACE/ACE-2 and Ang-II/angiotensin [28]. This makes ACE-2 a potential target for the development of specific treatments, antibodies, and vaccines related to COVID-19 infection. In this context, the HLA complex plays a substantial role in the association of genetic variability with the differential clinical outcomes of COVID-19. HLA-based approaches can be used as a molecular determinant tool to understand disease transmission and physiopathogenesis. This mini-review aims to shed light on the HLA-conferred differential susceptibility to COVID-19, antiviral immune responses, and the genetic comprehension of ACE-2 expression.

## 2. HLA Variants in COVID-19 Disease

**Figure 2:**
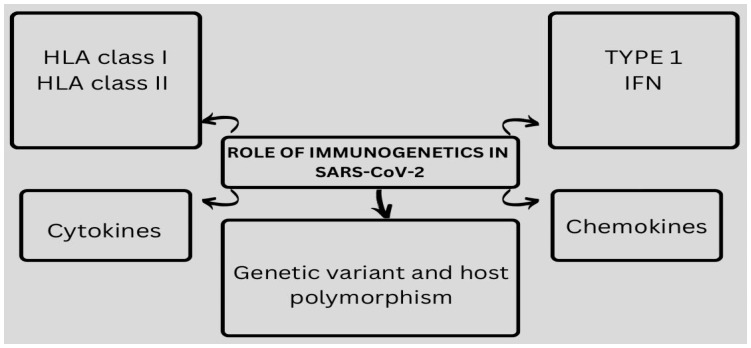
The role of immunogenetics in SARS-CoV-2 infection.

The influence of HLA variations on viral infections can provide insights into pathogenesis, identify at-risk individuals in diverse populations, and potentially lead to the development of new prophylactic and therapeutic interventions. The HLA gene family is among the most diverse in the human genome, comprising different classes, including HLA-I and HLA-II [29] (Figure 2).

Several HLA variants have been linked to COVID-19 risk and severity across various populations. HLA class I and class II alleles have also been associated with the severe acute respiratory syndrome caused by SARS-CoV [30,31,32]. Given its crucial role in the immune response, we hypothesize that variation in the HLA locus may provide a window into the mechanisms mediating COVID-19 infection. Recent in silico studies on HLA for peptide design can shed light on this topic [33,34].

A study by Wang et al. compared 82 COVID-19 patients to 3548 controls from China and identified HLA-B*15:27 as a statistically significant risk factor for SARS-CoV-2 infection (*p*-value 0.001, OR 3.6) [35]. Another recent study involving 190 patients and 423 controls found HLA-A*11:01:01:01 to be a risk factor for COVID-19 severity (*p*-value 0.003, OR 3.4) after controlling for comorbidities and other confounding factors [36]. In the immunogenetics section of Nature [37], researchers identified HLA-B*46:01 as the least expressing allele and HLA-B*15:03 as the most apparent allele, suggesting potential risk and protective factors for COVID-19 infections [37,38] (Table 1 and Table 2).

During the current pandemic, the rapid global spread of the SARS-CoV-2 virus led to various clinical and academic efforts to better comprehend the genetic interactions with the host and the severity of COVID-19. Understanding the impact of HLA variations in the disease holds significant promise in terms of understanding the immunopathogenesis of COVID-19, given its crucial role in the immunological response. Previous research has linked HLA polymorphisms to viral infections and their consequences, regardless of whether they confer protection or susceptibility [39]. As a result of the well-established significance of MHC/HLA polymorphism in infectious disease development and susceptibility, immunologists and geneticists have collaborated to investigate its function in SARS-CoV-2 infection and COVID-19 progression. The main goal of the COVID-19 HLA and Immunogenetics Consortium is to aid and unify these efforts. While previous studies have shown an association between HLA and SARS-CoV-2, additional research is still needed. In this review, we acknowledge recent findings linking HLA variation to COVID-19 and propose further advancements for these initiatives [40]. The effect of HLA on SARS-CoV-2 infection appears to be milder and primarily limited to severe symptoms, meaning its role still requires further research.

## 3. Angiotensin-Converting Enzyme 2 (ACE-2) in COVID-19

**Figure 3:**
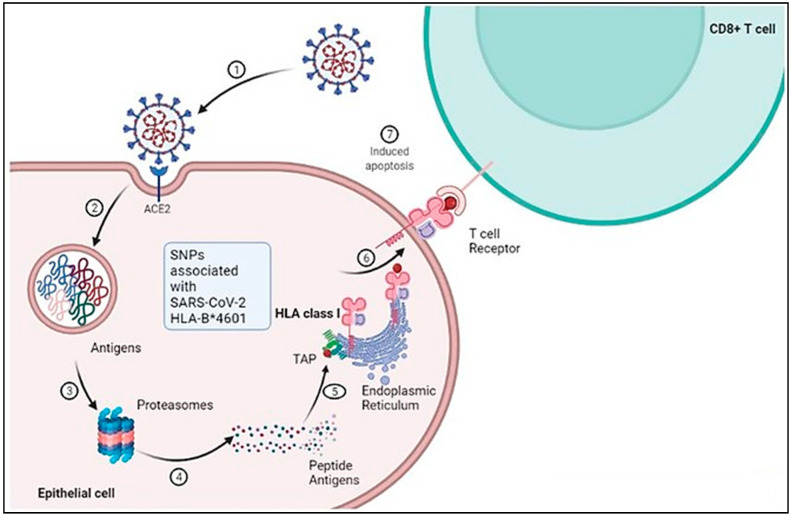
Schematic representation of cellular and molecular pathophysiology of SARS- CoV-2 (COVID-19).SARS-CoV-2 interacts with the ACE-2 receptor resulting in membrane fusion and cytoplasmic entry. 2 and 3: Proteasomal degradation of SARS-CoV-2 in the endolysosome results in antigen generation. 4 and 5: Further processing of the peptide antigens through interaction with HLA class 1 and complexing with the endoplasmic reticulum leads to its presentation on the cell surface. 6 and 7: The processed antigen is further represented by the APC through its β2M receptor, which activates T cells when they encounter the T cell receptors, leading to induced apoptosis. Adapted from “Acute Immune Responses to Coronaviruses”, by BioRender.com (2022). https://app.biorender.com/biorender-templates (accessed on 6 December 2022).

The susceptibility of the host to SARS-CoV-2 infection is determined by the expression of the cellular receptor ACE-2, which varies in different human tissues [41]. ACE-2 is highly expressed in adipose tissues, the heart, small intestine, thyroid, and testis, while it is moderately expressed in the lung, adrenal gland, bladder, colon, and liver. This partly explains the cardiac injuries observed in SARS-CoV-2 infected patients [42]. On the other hand, lower levels of ACE-2 expression have been reported in the spleen, bone marrow, and white blood cells [42]. The detection of SARS-CoV-2 in the stool of infected patients suggests that ACE-2 is expressed in the gastrointestinal tract [43]. The broad cellular tropism observed in SARS-CoV-2 infection is due to the wide range of predominant symptoms [42]. Although the expression levels of ACE-2 remain consistent in every individual, no significant gender, age, or race-based difference in expression has been reported so far [42]. There is a difference between the protein expression of ACE-2 and its mRNA expression level, indicating that ACE-2 is regulated post-transcriptionally [42] (Figure 3).

## 4. Genetic Polymorphism of ACE-2

Recently, genetic polymorphisms in ACE-2 have been reported in various cases, which can affect host susceptibility patterns by altering virus–host interactions. Various variants of ACE-2, such as S19P, T92I, I21V, K26R, E23K, T27A, Q102P, N64K, and H378R, have been found to increase the host’s susceptibility to COVID-19 [44]. In addition, ACE-2 expression correlates with different immune signatures, which in turn vary across different populations based on gender, age, and race. These factors can contribute to the high mortality rate associated with COVID-19 [45].

SARS-CoV-2 penetrates host cells via angiotensin-converting enzyme 2 (ACE-2), which is abundantly expressed in the heart, kidneys, and lungs and sheds into the plasma. ACE-2 regulates the renin-angiotensin-aldosterone system (RAAS). SARS-CoV-2 disrupts the ACE/ACE-2 balance and activates the RAAS, ultimately, leading to COVID-19 development, particularly in individuals with comorbidities [46,47]. The SARS-CoV-2 spike protein binds to ACE-2 on the cell surface, leading to the internalization and degradation of ACE-2 and a reduction in its expression. This shift in the balance between ACE and ACE-2, in favor of ACE, can result in increased angiotensin II production and activation of the RAAS, leading to the release of proinflammatory cytokines and contributing to the development of cardiovascular and renal complications in COVID-19.

**Figure 4:**
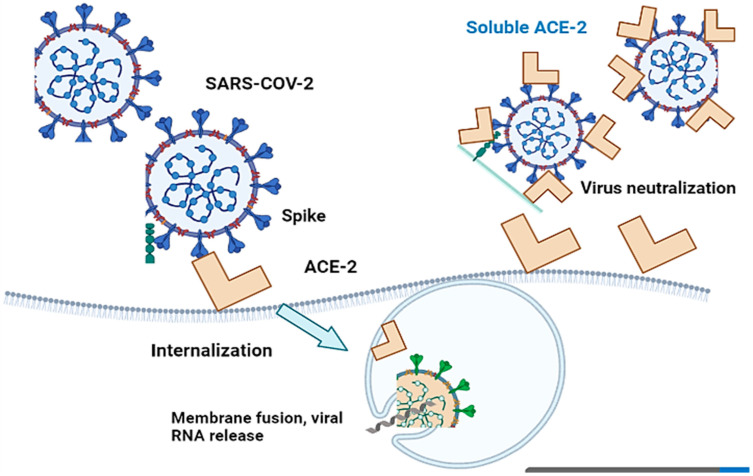
Cell entry of SARS-CoV-2 with ACE-2-mediation and virus infection inhibition by recombinant soluble ACE-2 protein. SARS-CoV-2 penetrates host cells via angiotensin-converting enzyme 2 (ACE-2), which subsequently results in the fusion of viral and cellular membranes.

Angiotensin-converting enzyme 2 (ACE-2) is critical for SARS-CoV-2 infection of a host species, as the S1 protein/receptor interaction is essential for obtaining access to host cells. S1 comprises a receptor-binding domain (RBD), which directly attaches to the peptidase domain (PD) in ACE-2 [48]. This is followed by cleavage of the S1 protein, which is achieved through acid-dependent proteolytic cleavage by one or several host proteases, including cathepsins, transmembrane serine protease (TMPRSS2), TMPRSS4, or human airway trypsin-like protease [49]. This results in the fusion of viral and cellular membranes. Following fusion with the host membrane, two heptad repeats in the S2 protein form a funnel-like shape in an antiparallel six-helix bundle, allowing fusion and release of the viral genome into the cytoplasm [50] (Figure 4). After replication and subgenomic RNA production, the viral structural proteins are translated and inserted into the endoplasmic reticulum (ER), and then, moved through the secretory route to the endoplasmic reticulum–Golgi intermediate, releasing multiple virions [51].

The crystal structures of the most powerful antibodies (P2C-1F11, P2B-2F6, and P2C-1A3) confirmed competition with ACE-2 binding, demonstrating that inhibiting the RBD and ACE-2 can mediate viral neutralization [52]. After screening a wide panel of human mAbs that target the spike protein, two antibodies, COV2-2196 and COV2-2130, were found to identify non-overlapping epitopes on the RBD and bound concurrently to the S protein, synergistically triggering viral neutralization [53].

## 5. The Association of Antiviral Immune Responses to COVID-19 Severity

Innate immunity serves as the body’s initial defense against viral infections, such as SARS-CoV-2, and is quickly followed by the expression of interferons, including alpha (α), beta (β), and lambda (λ). Cytokines released from the innate immune system and interferons further activate adaptive immunity, leading to the expression of various antiviral proteins [45]. However, severe infections initially downregulate the type 1 interferon, resulting in an unstable innate immune response [54]. In the case of SARS-CoV-2, higher levels of lambda interferon have been shown to decrease the clinical manifestations of COVID-19, as the viral replication is restricted by IFNL3/4 SNPs [55]. However, the lambda interferon response weakens over time in addition to a reduction in CD4+ T cell count, which leads to a weakened memory generation [45]. T cells can become exhausted during ongoing infections, with elevations in programmed cell death protein-1 (PD-1) and Tim-3 leading to lower protective T cell immunity [56]. In contrast, CD8+ T cells show clonal expansion in mild SARS-CoV-2 cases, while severe cases exhibit minimal expansion [54]. In some instances, younger individuals may develop severe COVID-19, even in the absence of comorbidities. This can be due to innate inaccuracies of immunity that alter the course of the infection, yet host genetic variation can also lead to an amplified or dysregulated immune response, resulting in more severe and life-threatening complications [54].

**Figure 5:**
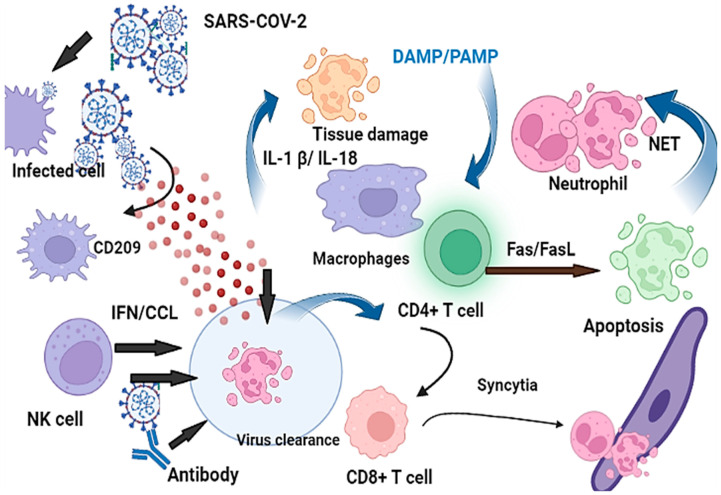
The cytokines secreted from the innate immune system counterparts and the interferons further trigger adaptive immunity leading to the expression of many types of antiviral proteins. Genes that play a key role include chemokines and their related receptors along with members of the IFN pathway. Dysregulated neutrophil extracellular traps (NET) formations persuade immune coagulation and intensify inflammation in the lungs of patients with COVID-19.

Genetic studies have suggested that genetic variations in CC-chemokine receptors, such as CCR2, are strongly associated with severe illnesses in patients. Furthermore, genetic differences in CXCR6 and CCR3 have been reported in mild or severe COVID-19 infections, indicating that at least some of these genetic differences contribute to critical illness [57]. Additionally, activated macrophages (M1) and monocytes produce proinflammatory cytokines, which recruit cytotoxic effector cells and release proinflammatory cytokines [58]. This process triggers an extreme and violent inflammatory cascade in the infected lungs, disrupting the endothelial, air–blood barrier, epithelial, and alveolar–epithelial barrier [59]. Insights into the immunopathogenesis and pathology of COVID-19 suggest that potential susceptibility genes, such as genes for inflammation, autoimmunity, cytokines, toll-like receptors (TLRs), and interleukins (IL1 and IL6) may be involved [42]. Consistently, neutrophilia and lymphopenia have been reported in COVID-19 patients, particularly those with severe complications from COVID-19 [43]. T cell deficiency, abnormal B cells, immune dysregulation, and potentially unnecessary production of IL-6 collectively contribute to the pathogenesis and severity of SARS-CoV-2 infections [41] (Figure 5).

## 6. The Role of HLA in Antiviral Immunity against SARS-CoV-2

One of the most critical determinants of disease progression in viral infections is the human leukocyte antigen (HLA) system. HLA molecules on the surface of cells have a crucial function in presenting endogenous and foreign antigens to T cells for identification and response [60]. HLA variation has been linked to multiple conditions and disorders, including infections, which are a significant cause of human mortality [61] and a crucial selective pressure that alters the human genome, particularly the HLAs [41]. The HLA system plays a vital role in the viral antigen presentation pathway, influencing differential viral susceptibility and disease severity. HLA inhibits pathogens, allowing the infected person to trigger an effective immune response against infection [62].

**Figure 6:**
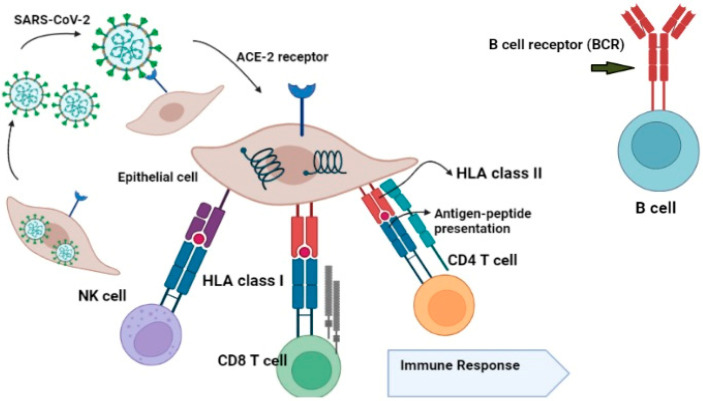
B cell receptor (BCR) proteins are present at B cells receptors, while T cell receptor (TCR) proteins are only found on the surface of T cells. When the TCR identifies the HLA molecule complex bonded with the foreign peptide, it activates the immune system to be aware of the presence of a foreign protein. Activated T cells can kill infected cells, or activate B cells, which produce antibodies in response to an infection.

Genetic variation in the three MHC class I genes (human leukocyte antigen A (HLA-A), -B, and -C genes) may influence susceptibility to, and intensity of, the disease caused by severe acute respiratory syndrome coronavirus-2 (SARS-CoV-2) [24]. Several classical transmembrane proteins are encoded by HLA genes, such as HLA-A, HLA-B, HLA-C, HLA-DR, HLA-DQ, and HLA-DP and are predominantly associated with the presentation of antigens on the surface of cells that further trigger antiviral responses [26]. For instance, HLA-B*01 and/or HLA-B*44 are usually found in healthy people, while individuals with HLA-B*08 and HLA-A*25 are at higher risk of COVID-19 infections, as they lack the immune-dominant virus-derived epitope peptides and cannot trigger sufficient immune responses [63]. Moreover, HLA-C*01 and/or HLA-B*44 were also reported to have an association with inflammatory autoimmune diseases from SARS-CoV-2 infection and inappropriate immunological reactions [64]. It has been reported that HLA haplotypes/alleles are associated with stronger immune responses to viral infections. Several polymorphisms have been reported to influence the SARS-CoV virus susceptibility, including HLA-B*07:03, HLA-Cw*08:01, HLADRB1*12:02, and HLA-B*46:01 (Figure 1) [31]. Various alleles, such as HLA-A*02:01, HLA-B*08:01, HLAC*07:01, and HLA-B*18:01, have the potential to present viral peptides [24]. In addition, the T cell-mediated immune response depends on the individuals’ HLA profiles, as people with different profiles respond differently to the same antigen [65]. Based on binding affinity prediction studies, SARS-CoV-2 is predominantly presented by the HLA-A allele compared to its HLA-C counterpart [64] (Figure 6).

## 7. Mechanism of HLA Alleles

Previous studies have investigated the significance of HLA molecules in immune modulation in persistent viral infections. HLA loci were discovered in the 1970s and have since been identified as genetically determined candidates for infectious disease predisposition [66]. HLAs are classified as major histocompatibility complexes (MHCs) due to their importance in allowing the immune system to distinguish “self” from “non-self” antigens. Cells have a range of barrier systems to protect themselves, and the immune system closely monitors the interior environment of cells using the MHC. Cell metabolism degrades old or unneeded proteins on a regular basis, and fragments of these metabolites are displayed on the cell surface. MHC binds to these peptides, enabling the immune system to recognize and analyze them [67]. There are multiple antigenic types of HLA antigens, similar to MHC in other species, such as mice. In humans, class Ia (HLA-A, -B, and -C), class Ib (HLA-E, -F, -G, and -H), and class II (HLA-DR, -DQ, -DM, and -DP) HLA loci are involved in antigen presentation to CD8+ T cells, natural killer cells (NK cells), and CD4+ T cells, respectively [68,69]. However, NK cells also recognize both class Ia and Ib molecules. MHC class I molecules are found on almost all nucleated cells and platelets and primarily serve to present endogenous antigens [70]. MHC class I molecules are classified into classical and nonclassical groups. The classical class I molecules present antigens to T cells, while the nonclassical molecules have limited polymorphisms and serve a wide range of functions. The peptides presented by MHC class I molecules through T cell receptors (TCRs) are obtained from endogenous protein antigens created by the proteolysis of non-self-cells, such as virus-infected cells or tumor cells. These processed antigens are delivered to the MHC class I molecules through the peptide-binding cleft. MHC class I molecules bind peptides ranging in length from 8 to 11 amino acids [71].

MHC class II molecules play a crucial role in presenting exogenous antigen-derived peptides. These peptides are produced by the lysosomal breakdown of exogenous foreign target cells, such as bacteria or fungi, which have entered the cell via endocytosis [72]. When MHC class II molecules present antigens on the peptide-binding cleft and are recognized by CD4+ T cells through the TCR, the CD4+ T cells become activated and release cytokines that stimulate both Th1 and Th2 cells [16]. Then, Th1 cells release IL-2 and IFN-γ, which activate CD8+ cytotoxic T cells and natural killer cells (NK) [73]. The alleles of HLA influence the T cell immune response by presenting various peptide fragments of invading pathogens [74]. HLA proteins play a significant role in establishing human immunity by controlling a range of destined epitopes [75]. The HLA has the potential to recognize any foreign antigens by T cells. In the establishment of adaptive immune responses to viral antigens, the key role played by HLA is the presentation of viral antigens by APC cells, as well as the direct presentation of HLA I to cytotoxic CD8+ T cells [64]. The HLA subtypes, such as HLA-DQN1*04 and HLA-DRBI*04, determine the severity of infections or pathogenicity, as has been shown for the hepatitis C virus, human immunodeficiency virus, human papillomavirus (HPV), and human hepatitis B virus [76].

HLA/MHC class I molecules, specifically HLA-A, HLA-B, and HLA-C, present intracellular antigens, such as viral or tumor antigens, to CD8+ T cells (cytotoxic T lymphocytes or CTLs) and natural killer (NK) cells, leading to a cytotoxic immune response [77]. When a cell is infected by a virus, protein fragments of the virus are generated through proteasomal digestion and presented on the surface of the cell by the HLA system to be recognized and eliminated by the immune system. Typically, these peptides are small polymers consisting of nine amino acids. The CTLs recognize the HLA-peptide complex through their T cell receptor. On the other hand, HLA/MHC class II molecules induce a helper T cell response by presenting extracellular antigens to CD4+ lymphocytes. This response reinforces the activation of CD8+ lymphocytes and establishes long-term memory [78]. In addition, helper T lymphocytes aid in the production of neutralizing antibodies against the specific antigen by B lymphocytes.

**Figure 7:**
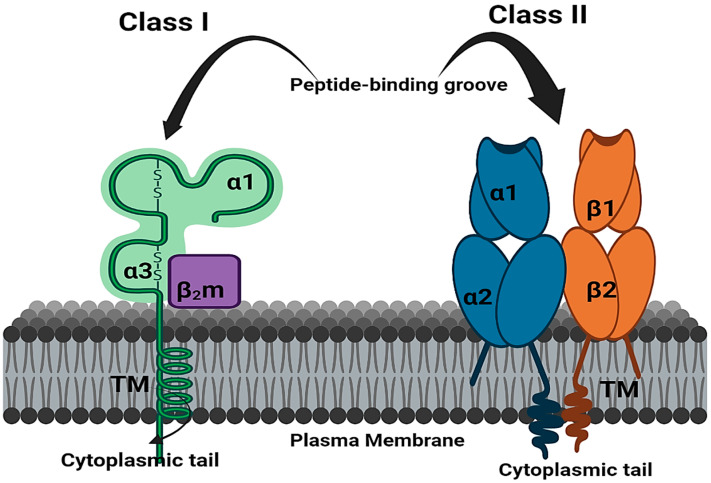
Schematic structure of HLA class I and class II molecules. The peptide-binding groove in class I and class II molecules are important for functional aspects of HLA molecule. Class I antigens consist of two chains: a glycoprotein heavy chain and a β2-microglobulin molecule. Class II consists of two structurally similar α- and β-glycoprotein chains, each chain has two amino acid domains, of which the outermost domain contains the variable region of class II alleles.

Viruses are intracellular antigens that can be subjected to proteolytic digestion in the proteasome. In the endoplasmic reticulum, HLA class I molecules are bounded by these antigenic peptides. However, intracellular, and extracellular antigens are processed by class I and II HLA molecules, respectively. Then, the HLA-peptide complexes are transferred to the cell membrane, where class I molecules are expressed in abundance, while class II molecules are expressed by cells specialized for antigen presentation, such as dendritic cells, monocytes, macrophages, and B lymphocytes [79] (Figure 7).

## 8. The Regional Analysis of HLA

Every individual who has an ACE-2 receptor is susceptible to COVID-19. However, host susceptibility is also influenced by genetic variations in the HLA genes, which encode hundreds of protein-coding genes that regulate fundamental molecular and cellular processes, including immune responses [26]. The HLA genes are part of the major histocompatibility complex (MHC) located on the short arm of chromosome 6, which is one of the most complex genetic systems. Historically, the etiological agents of infectious diseases have been traced using the HLA system, and disease outcomes have been predicted by their interactions [19]. Very few studies have evaluated the influence of HLA haplotypes on SARS-CoV-2 infection susceptibility and severity, although some inexplicable differences in SARS-CoV-2 infection severity and mortality rates have been identified by HLA haplotypes worldwide [80]. Previously, in Taiwan, HLA-B*46:01 has been found to be associated with SARS-CoV infection, in contrast to the predominant HLA-A haplotype used by the virus. The HLA alleles strongly mediated the viral antigen presentation pathway, which is believed to be a critical component in COVID-19 susceptibility and severity. It has been observed that individuals with the HLA-B*46:01 genotype had a higher susceptibility and severity of COVID-19 [81].

## 9. HLA Variants in the Genetic Susceptibility

**Figure 8:**
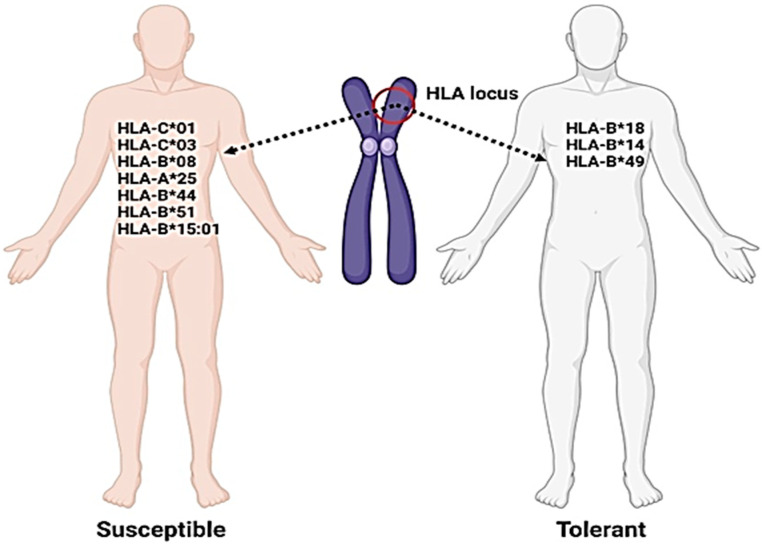
Schematic representation of individuals harboring susceptible and tolerant HLA variants. Created with BioRender.com (2022). Susceptible organisms have higher chances of COVID-19 infection and the tolerant have fewer chances.

In addition to age, gender, and health status, genetic variants of HLA have been found to influence susceptibility to various viral diseases, including SARS-CoV, MERS-CoV, influenza, dengue, and hepatitis B. Susceptibility to and severity of COVID-19 are also considerably associated with genetic variation in HLA, which plays a significant role in identifying populations at higher risk of COVID-19 disease [26]. HLA molecules of classes I/II play a significant role in human immunity and determine individual susceptibility to COVID-19 infection. Certain alleles, such as HLA-C*01C*03, B*08, A*25, B*44, B*51, and B*15:01, have been positively correlated with the incidence rate of COVID-19, while other alleles, such as HLA-B*18, B*14, and B*49, have shown negative correlation (Figure 8) [65]. Notably, the incidence of COVID-19 is not influenced by any single allele. Haplotypes and HLA-specific alleles could be used as parameters to provide clues about highly susceptible populations to COVID-19 [31]. Across countries, HLA may be the most suitable marker for evaluating COVID-19 susceptibility in epidemic tendencies. HLA association with COVID-19 susceptibility and epidemic situations can have similar patterns across different countries, which are clearly explained by HLA polymorphisms [26].

## 10. HLA-Based Vaccines

Various allelotypes, such as HLA-A*02:01, HLA-DR*03:01, and HLA-Cw*15:02, have been reported to protect against SARS infections [60] (Table 3). In vaccine development, the consequential epitopes of B and T cells of SARS-CoV-2, and their association with HLA alleles, can contribute to the development of protective antibodies against SARS-CoV-2 infection [82]. HLA-C*01 and HLA-B*44 are potential molecular determinants in evaluating an individual’s risk of COVID-19, and genotyping of class I and II HLA in COVID-19 patients can identify individuals at a higher risk of a cytokine storm [62]. The HLA-C*01 and B*44 allele pathways can significantly aid in understanding disease transmission, physiopathogenesis, and play a significant role in managing COVID-19 disease, vaccination, and other preventive strategies in terms of clinical management [62]. Peptide cocktail vaccines produced by using various epitopes constrained by the HLA-A2 molecule provoke substantial specific CD8+ T cell responses against SARS-CoV-2 in experimental animals [83]. Additionally, this reduces the pathological changes in the lungs of mice. Although the exact mechanism of such specific responses remains unknown [31].

After the discovery of some promising natural compounds [7,84,85] with significant antiviral activity against COVID-19, the scientific focus has been directed toward host-directed therapies, such as HLA-based treatments.

**Table 3:** Host HLA allelic interactions with SARS-CoV-2 Infection during pathogenesis.

Human Leukocyte Antigens	Association with SARS-CoV Infections	References
HLA-B*07:03, HLA-Cw*08:01, HLADRB1*12:02, HLA-B*46:01	Increase the susceptibility	[86,87]
HLA-A*02:01, HLA-DR*03:01, and HLA-Cw*15:02	Provide protection against SARS-CoV-2	[24]
HLA-C*01 and/or HLA-B*44	Have association with inflammatory autoimmune diseases in SARS-CoV-2 infection and inappropriate immunological reaction	[88]
HLA-C*01 and B*44 alleles	Can significantly help in the understanding of disease transmission and physiopathogenesis	[89]

## 11. Concluding Remarks

This review provides genetic insights into the clinical outcomes and disease severity of COVID-19 infections. The association of ACE-2 with immune signatures significantly affects clinical outcomes and mortality rates. The review also explores the relationship between HLA and COVID-19 infection, offering the genetic basis for variations in infection outcomes. HLA-C*01 and HLA-B*44 are potential molecular determinants for evaluating an individual’s risk of COVID-19. Genotyping of class I and II HLA in COVID-19 patients can identify those at higher risk of a cytokine storm. HLA can be a suitable marker for evaluating COVID-19 susceptibility in epidemic tendencies globally. The paths of HLA-C*01 and B*44 alleles can significantly aid in understanding disease transmission and physiopathogenesis. HLA alleles influence T cell immune responses by presenting various peptide fragments of invading pathogens. Peptide cocktail vaccines produced by using various epitopes constrained by the HLA-A2 molecule provoked substantial specific CD8+ T cell responses in experimental mice and reduced pathological changes in the lungs, although the exact mechanism remains unknown.

## Figures and Tables

**Table 1:** HLA nomenclature basis: alleles and their descriptions.

Alleles	Description	Reference
HLA	Refers to the HLA region and prefix for an HLA gene	[38]
HLA-DRB1*13	Refers to all alleles in the DR13 serologic group	
HLA-DRB1*13:01	Refers to a specific HLA allele	
HLA-DRB1*13:01:02	Refers to an allele that differs by a synonymous mutation from DRB1*13:01:01	
HLA-DRB1*13:01:01:02	Refers to an allele that contains a mutation outside the coding region from DRB1*13:01:01:01	
HLA-DRB1	Refers to a particular HLA locus, i.e., DRB1	
HLA-A*24:02:01:02L	Refers to an allele encoding a protein with significantly reduced or ‘low’ cell surface expression, where the mutation is found outside the coding region	
HLA-B*44:02:01:02S	Refers to an allele encoding a protein that is expressed as a ‘secreted’ molecule only	
HLA-A*32:11Q	Refers to an allele that has a mutation that has previously been shown to have a significant effect on cell surface expression, although this has not been confirmed and its expression remains ‘questionable’	
HLA-A*24:09N	Refers to a ‘null’ allele, an allele that is not expressed	
HLA-A*30:14L	Refers to an allele encoding a protein with significantly reduced or ‘low’ cell surface expression.	

**Table 2:** Numbers of recognized isolated antigen specificities and alleles, variants, or subtypes of HLA class I and class II antigens.

Locus	Antigen Specificities	Alleles	Reference
HLA-A	24	303	[38]
HLA-B	55	559	
HLA-C	9	150	
HLADRB1	17	362	
Total	105	1374	

## Data Availability

Not applicable.

## References

[1] Ul Haq I., Krukiewicz K., Yahya G., Haq M.U., Maryam S., Mosbah R.A., Saber S., Alrouji M. (2023). The breadth of bacteriophages contributing to the development of the phage-based vaccines for COVID-19: An ideal platform to design the multiplex vaccine. Int. J. Mol. Sci..

[2] Egbuna C., Chandra S., Awuchi C.G., Aklani S., Ulhaq I., Akram M., Khan J. (2022). Myth surrounding the FDA disapproval ofhydroxychloroquine sulfate and chloroquine phosphate as drugs for coronavirus disease 2019. Coronavirus Drug Discovery.

[3] Maryam S., Ul Haq I., Yahya G., Ul Haq M., Algammal A.M., Saber S., Cavalu S. (2023). COVID19 surveillance in wastewater: An epidemiological tool for the monitoring of SARS-CoV-2. Front. Cell. Infect. Microbiol..

[4] Ulhaq I., Basit A., Ali I., Hussain F., Ali Z., Siddique F., Ahmed H., Aqib A.I., Rahim K. (2021). Coronavirus Disease-2019 (COVID-19) Epidemiology. COVID-19: Epidemiology, Biochemistry, and Diagnostics.

[5] Basit A., Ulhaq I., Hussain F., Ud-Din Z., Rahim K. (2021). Nucleic Acid Based Detection of COVID-19. COVID-19: Epidemiology, Biochemistry, and Diagnostics.

[6] Ayaz M.M., Ulhaq I., Rahim k. (2021). Histopathologic Evaluation and Scoring of SARS-CoV-2 Infection. COVID-19: Different Models and Treatment Strategies.

[7] Ali I., Rasheed M.A., Cavalu S., Rahim K., Ijaz S., Yahya G., Goh L.P.W., Popoviciu M.S. (2023). Identification of Natural Lead Compounds against Hemagglutinin-Esterase Surface Glycoprotein in Human Coronaviruses Investigated via MD Simulation, Principal Component Analysis, Cross-Correlation, H-Bond Plot and MMGBSA. Biomedicines.

[8] Haq I.U., Khan Z.I., Aziz I., Basit A., Hussain F., Bibi A., Aqib A.I., Younas U., Rahim K. (2023). Chapter 12—Phages and SARS-CoV-2. Application of Natural Products in SARS-CoV-2.

[9] Metwally K., Abo-Dya N.E., Alahmdi M.I., Albalawi M.Z., Yahya G., Aljoundi A., Salifu E.Y., Elamin G., Ibrahim M.A.A., Sayed Y. (2023). The Unusual Architecture of RNA-Dependent RNA Polymerase (RdRp)’s Catalytic Chamber Provides a Potential Strategy for Combination Therapy against COVID-19. Molecules.

[10] http://covid19.who.int/.

[11] El-Sokkary M.M., El-Baz A.M., El-Morsi R.M., Keuper K., El-Hawary S., Shata A., Yahya G. (2022). Early forecasting of COVID-19 case progression with hematological and biochemical parameters of patients in Egypt. Paki. J. Pharma. Sci..

[12] Al Naggar Y., Giesy J.P., Abdel-Daim M.M., Javed Ansari M., Al-Kahtani S.N., Yahya G. (2021). Fighting against the second wave of COVID-19: Can honeybee products help protect against the pandemic?. Saudi. J. Biol. Sci..

[13] Elmorsy M.A., El-Baz A.M., Mohamed N.H., Almeer R., Abdel-Daim M.M., Yahya G. (2022). In silico screening of potent inhibitors against COVID-19 key targets from a library of FDA-approved drugs. Environ. Sci. Pollut. Res..

[14] Mostafa I., Mohamed N.H., Mohamed B., Almeer R., Abulmeaty M.M.A., Bungau S.G., El-Shazly A.M., Yahya G. (2022). In-silico screening of naturally derived phytochemicals against SARS-CoV Main protease. Environ. Sci. Pollut. Res..

[15] Shaldam M.A., Yahya G., Mohamed N.H., Abdel-Daim M.M., Al Naggar Y. (2021). In silico screening of potent bioactive compounds from honeybee products against COVID-19 target enzymes. Environ. Sci. Pollut. Res..

[16] Yahya G., Mansour B., Keuper K., Shaldam M., El-Baz A.M. (2021). Virtual Screening Attributes Male Biased COVID-19 Mortality to Predicted Antiviral Activity of Female Sex Hormones. Let. Drug Des. Discov..

[17] Al-Karmalawy A.A., Soltane R., Abo Elmaaty A., Tantawy M.A., Antar S.A., Yahya G., Chrouda A., Pashameah R.A., Mustafa M., Abu Mraheil M. (2021). Coronavirus Disease (COVID-19) Control between Drug Repurposing and Vaccination: A Comprehensive Overview. Vaccines.

[18] Lan N.T.P., Kikuchi M., Huong V.T.Q., Ha D.Q., Thuy T.T., Tham V.D., Tuan H.M., Van Tuong V., Nga C.T.P., Van Dat T. (2008). Protective and Enhancing HLA Alleles, HLA-DRB1*0901 and HLA-A*24, for Severe Forms of Dengue Virus Infection, Dengue Hemorrhagic Fever and Dengue Shock Syndrome. PLoS Negl. Trop. Dis..

[19] Lin M., Tseng H.-K., Trejaut J.A., Lee H.-L., Loo J.-H., Chun-Hsiung H., Chen P.-J., Su Y.-W., Lim K.H., Tsai Z.-U. (2003). Association of HLA class I with severe acute respiratory syndrome coronavirus infection. BMC. Med. Gen..

[20] Hajeer A.H., Balkhy H., Johani H., Yousef M., Arabi Y. (2016). Association of human leukocyte antigen class II alleles with severe Middle East respiratory syndrome—Coronavirus infection. Ann. Thorac. Med..

[21] Luckey D., Weaver E.A., Osborne D.G., Billadeau D.D., Taneja V. (2019). Immunity to Influenza is dependent on MHC II polymorphism: Study with 2 HLA transgenic strains. Sci. Rep..

[22] Nishida N., Ohashi J., Khor S.-S., Sugiyama M., Tsuchiura T., Sawai H., Hino K., Honda M., Kaneko S., Yatsuhashi H. (2016). Understanding of HLA-conferred susceptibility to chronic hepatitis B infection requires HLA genotyping-based association analysis. Sci. Rep..

[23] Shiina T., Hosomichi K., Inoko H., Kulski J.K. (2009). The HLA genomic loci map: Expression, interaction, diversity and disease. J. Hum. Genet..

[24] Nguyen A., David J.K., Maden S.K., Wood M.A., Weeder B.R., Nellore A., Thompson R.F. (2020). Human leukocyte antigen susceptibility map for severe acute respiratory syndrome coronavirus 2. J. Virol..

[25] Savage S.A., Viard M., O’Huigin C., Zhou W., Yeager M., Li S.A., Wang T., Ramsuran V., Vince N., Vogt A. (2020). Genome-wide association study identifies HLA-DPB1 as a significant risk factor for severe aplastic anemia. Am. J. Hum. Gen..

[26] Patel N., Sethi Y., Kaka N., Kaiwan O., Gupta I., Shaheen R.S., Sapoor S., Chopra H., Popoviciu M.S., Emran T.B. (2023). Acute Hepatitis of Unknown Origin in Pediatric Age Group: Recent Outbreaks and Approach to Management. J. Clin. Med..

[27] Ni W., Yang X., Yang D., Bao J., Li R., Xiao Y., Hou C., Wang H., Liu J., Yang D. (2020). Role of angiotensin-converting enzyme 2 (ACE2) in COVID-19. Crit. Care.

[28] Rodrigues R., de Oliveira S.C. (2021). The Impact of Angiotensin-Converting Enzyme 2 (ACE2) Expression Levels in Patients with Comorbidities on COVID-19 Severity: A Comprehensive Review. Microorganisms.

[29] Jin Y., Wang J., Bachtiar M., Chong S.S., Lee C.G.L. (2018). Architecture of polymorphisms in the human genome reveals functionally important and positively selected variants in immune response and drug transporter genes. Hum. Genom..

[30] Keicho N., Itoyama S., Kashiwase K., Phi N.C., Long H.T., Van Ban V., Hoa B.K., Le Hang N.T., Hijikata M., Sakurada S. (2009). Association of human leukocyte antigen class II alleles with severe acute respiratory syndrome in the Vietnamese population. Hum. Immunol..

[31] Reusch N., De Domenico E., Bonaguro L., Schulte-Schrepping J., Babler K., Schultze J.L., Aschenbrenner A.C. (2021). Neutrophils in COVID-19. Front. Immunol..

[32] Tavasolian F., Rashidi M., Hatam G.R., Jeddi M., Hosseini A.Z., Mosawi S.H., Abdollahi E., Inman R.D. (2021). HLA, Immune Response, and Susceptibility to COVID-19. Front. Imunol..

[33] Augusto D.G., Hollenbach J.A. (2022). HLA variation and antigen presentation in COVID-19 and SARS-CoV-2 infection. Curr. Opin. Immunol..

[34] Abbasifard M., Khorramdelazad H. (2020). The bio-mission of interleukin-6 in the pathogenesis of COVID-19: A brief look at potential therapeutic tactics. Life Sci..

[35] Wang W., Zhang W., Zhang J., He J., Zhu F. (2020). Distribution of Allele Frequencies in 82 Chinese Individuals with Coronavirus Disease-2019 (COVID-19). HLA.

[36] Khor S.-S., Omae Y., Nishida N., Sugiyama M., Kinoshita N., Suzuki T., Suzuki M., Suzuki S., Izumi S., Hojo M. (2021). HLA-A*11:01:01:01, HLA-C*12:02:02:01-HLA-B*52:01:02:02, Age and Sex Are Associated with Severity of Japanese COVID-19 with Respiratory Failure. Front. Immunol..

[37] Zahn L.M. (2020). HLA Genetics and COVID-19. Science.

[38] http://hla.alleles.org/nomenclature/naming.html.

[39] Douillard V., Castelli E.C., Mack S.J., Hollenbach J.A., Gourraud P.A., Vince N., Limou S., Covid-19|HLA & Immunogenetics Consortium and the SNP-HLA Reference Consortium (2021). Current HLA Investigations on SARS-CoV-2 and Perspectives. Front. Genet..

[40] Astbury S., Reynolds C.J., Butler D.K., Muñoz-Sandoval D.C., Lin K.M., Pieper F.P., Otter A., Kouraki A., Cusin L., Nightingale J. (2022). HLA-DR polymorphism in SARS-CoV-2 infection and susceptibility to symptomatic COVID-19. Immunology.

[41] Li M., Li L., Zhang Y., Wang X.S. (2020). Expression of the SARS-CoV-2 cell receptor gene ACE2 in a wide variety of human tissues. Infect. Dis. Poverty.

[42] Diao B., Wang C., Tan Y., Chen X., Liu Y., Ning L., Chen L., Li M., Liu Y., Wang G. (2020). Resuction and functional exhaustion of T cells in patients with Coronavirus Disease COVID-19. Front. Immunol..

[43] Xu J., Chu M., Zhong F., Tan X., Tang G., Mai J., Lai N., Guan C., Liang Y., Liao G. (2020). Digestive symptoms of COVID-19 and expression of ACE2 in digestive tract organs. Cell Death. Discov..

[44] Suryamohan K., Diwanji D., Stawiski E.W., Gupta R., Miersch S., Liu J., Chen C., Jiang Y.-P., Fellouse F.A., Sathirapongsasuti J.F. (2021). Human ACE2 receptor polymorphisms and altered susceptibility to SARS-CoV-2. Commun. Biol..

[45] Chen N., Zhou M., Dong X., Qu J., Gong F., Han Y., Qiu Y., Wang J., Liu Y., Wei Y. (2020). Epidemiological and clinical characteristics of 99 cases of 2019 novel coronavirus pneumonia in Wuhan, China: A descriptive study. Lancet.

[46] Beyerstedt S., Casaro E.B., Rangel É.B. (2021). COVID-19: Angiotensin-converting enzyme 2 (ACE2) expression and tissue susceptibility to SARS-CoV-2 infection. Eur. J. Clin. Microbiol. Infect. Dis..

[47] Tipnis S.R., Hooper N.M., Hyde R., Karran E., Christie G., Turner A.J. (2000). A human homolog of angiotensin-converting enzyme. J. Biol. Chem..

[48] Yan R., Zhang Y., Li Y., Xia L., Guo Y., Zhou Q. (2020). Structural basis for the recognition of SARS-CoV-2 by full-length human ACE2. Science.

[49] Hoffmann M., Kleine-Weber H., Schroeder S., Krüger N., Herrler T., Erichsen S., Schiergens T.S., Herrler G., Wu N.-H., Nitsche A. (2020). SARS-CoV-2 cell entry depends on ACE2 and TMPRSS2 and is blocked by a clinically proven protease inhibitor. Cell.

[50] Zhu L., She Z.-G., Cheng X., Qin J.-J., Zhang X.-J., Cai J., Lei F., Wang H., Xie J., Wang W. (2020). Association of blood glucose control and outcomes in patients with COVID-19 and pre-existing type 2 diabetes. Cell Metab..

[51] Fehr A.R., Perlman S. (2015). Coronaviruses: An overview of their replication and pathogenesis. Coronaviruses.

[52] Ju B., Zhang Q., Ge J., Wang R., Sun J., Ge X., Yu J., Shan S., Zhou B., Song S. (2020). Human neutralizing antibodies elicited by SARS-CoV-2 infection. Nature.

[53] Zost S.J., Gilchuk P., Case J.B., Binshtein E., Chen R.E., Nkolola J.P., Schäfer A., Reidy J.X., Trivette A., Nargi R.S. (2020). Potently neutralizing and protective human antibodies against SARS-CoV-2. Nature.

[54] Carter-timofte M.E., Jørgensen S.E., Freytag M.R. (2020). Deciphering the Role of Host Genetics in Susceptibility to Severe COVID-19. Front. Immunol..

[55] Rahimi P., Tarharoudi R., Rahimpour A., Amroabadi J.M., Ahmadi I., Anvari E., Siadat S.D. (2021). The association between interferon lambda 3 and 4 gene single-nucleotide polymorphisms and the recovery of COVID-19 patients. Virol. J..

[56] Zheng H., Zhang M., Yang C., Zhang N., Wang X.C., Yang X.P., Dong X.Q. (2020). Elevated exhaustion levels and reduced functional diversity of T cells in peripheral blood may predict severe progression in COVID-19 patients. Cell. Mol. Immunol..

[57] McCoy K., Peterson A., Tian Y., Sang Y. (2020). Immunogenetic Association Underlying Severe COVID-19. Vaccines.

[58] Duque G.A., Descoteaux A. (2014). Macrophage cytokines: Involvement in immunity and infectious diseases. Front. Immunol..

[59] Mozafari N., Azadi S., Mehdi-Alamdarlou S., Ashrafi H., Azadi A. (2020). Inflammation: A bridge between diabetes and COVID-19, and possible management with sitagliptin. Med. Hypoth..

[60] Ryan S.O., Cobb B.A. (2012). Roles for major histocompatibility complex glycosylation in immune function. Semin. Immunopathol..

[61] Burgner D., Jamieson S.E., Blackwell J.M. (2006). Genetic susceptibility to infectious diseases: Big is beautiful, but will bigger be even better?. Lancet Infect. Dis..

[62] Loi E., Moi L., Cabras P., Arduino G., Costanzo G., Del Giacco S., Erlich H.A., Firinu D., Caddori A., Zavattari P. (2022). HLA-C dysregulation as a possible mechanism of immune evasion in SARS-CoV-2 and other RNA-virus infections. Front. Immunol..

[63] Littera R., Campagna M., Deidda S., Angioni G., Cipri S., Melis M., Firinu D., Santus S., Lai A., Porcella R. (2020). Human Leukocyte Antigen Complex and Other Immunogenetic and Clinical Factors Influence Susceptibility or Protection to SARS-CoV-2 Infection and Severity of the Disease Course. Sard. Exp..

[64] Pisanti S., Deelen J., Gallina A.M., Caputo M., Citro M., Abate M., Sacchi N., Vecchione C., Martinelli R. (2020). Correlation of the two most frequent HLA haplotypes in the Italian population to the differential regional incidence of COVID-19. J. Transl. Med..

[65] Migliorini F., Torsiello E., Spiezia F., Oliva F., Tingart M., Maffuli M. (2021). Association between HLA genotypes and COVID-19 susceptibility, severity and progression: A comprehensive review of the literature. Eur. J. Med. Res..

[66] Silvestr D., Kourilsk F.M., Niccolai M.G., Levy J.P. (1970). Presence of HLA antigens on human reticulocytes as demonstrated by electron microscopy. Nature.

[67] Ujvari B., Belov K. (2011). Major Histocompatibility Complex (MHC) Markers in Conservation Biology. Int. J. Mol. Sci..

[68] Allard M., Oger R., Benlalam H., Florenceau L., Echasserieau K., Bernardeau K., Labarrière N., Lang F., Gervois N. (2014). Soluble HLA-I/peptide monomers mediate antigen-specific CD8 T cell activation through passive peptide exchange with cell-bound HLA-I molecules. J Immunol..

[69] Leddon S.A., Sant A.J. (2010). Generation of MHC class II-peptide ligands for CD4 T-cell allorecognition of MHC class II molecules. Curr. Opin. Organ Transplant..

[70] Gabriel C., Furst D., Fae I., Wenda S., Zollikofer C., Mytilineos J., Fischer G.F. (2014). HLA typing by next-generation sequencing-getting closer to reality. Tissue Antigens.

[71] Lundegaard C., Lamberth K., Harndahl M., Buus S., Lund O., Nielsen M. (2008). NetMHC-3.0: Accurate web accessible predictions of human, mouse and monkey MHC class I affinities for peptides of length 8–11. Nucleic Acids Res..

[72] Sadegh-Nasseri S., Kim A. (2015). Exogenous antigens bind MHC class II first and are processed by cathepsins later. Mol. Immunol..

[73] Fehniger T.A., Cooper M.A., Nuovo G.J., Cella M., Facchetti F., Colonna M., Caligiuri M.A. (2003). CD56bright natural killer cells are present in human lymph nodes and are activated by T cell-derived IL-2: A potential new link between adaptive and innate immunity. Blood.

[74] Kachuri L., Francis S.S., Morrison M.L., Wendt G.A., Bosse Y., Cavazos T.B., Rashkin S.R. (2020). The landscape of host genetic factors involved in immune response to common viral infections. Genome Med..

[75] Grifoni A., Sidney J., Vita R., Peters B., Crotty S., Weiskopf D., Sette D. (2021). SARS-CoV-2 human T cell epitopes: Adaptive immune response against COVID-19. Cell Host Microbe.

[76] Mai H., Chen J., Chen H., Liu Z., Huang G., Wang J., Xiao Q., Ren W., Zhou B. (2021). Fine Mapping of the MHC Region Identifies Novel Variants Associated with HBV-Related Hepatocellular Carcinoma in Han Chinese. J. Hepatocell. Carcinoma.

[77] Wieczorek M., Abualrous E.T., Sticht J., Álvaro-Benito M., Stolzenberg S., Noé F., Freund C. (2017). Major histocompatibility complex (MHC) class I and MHC class II proteins: Conformational plasticity in antigen presentation. Front. Immunol..

[78] Sahin Tekin M., Yorulmaz G., Yantir E., Gunduz E., Colak E. (2022). A Novel Finding of an HLA Allele’s and a Haplotype’s Relationship with SARS-CoV-2 Vaccine-Associated Subacute Thyroiditis. Vaccines.

[79] Kuznetsov A., Voronina A., Govorun V., Arapidi G. (2020). Critical Review of Existing MHC I Immunopeptidome Isolation Methods. Molecules.

[80] Lassale C., Gaye B., Hamer M., Galle C.R., Batty G.D. (2020). Ethnic disparities in hospitalization for COVID-19 in England: The role of socioeconomic factors, mental health, and inflammatory and pro-inflammatory factors in a community-based cohort study. Brain Behav. Immun..

[81] Li W., Moore M.J., Vasilieva N., Sui J., Wong S.K., Berne M.A., Somasundaran M., Sullivan J.L., Luzuriaga K., Greenough T.C. (2003). Angiotensin-converting enzyme 2 is a functional receptor for the SARS coronavirus. Nature.

[82] Ahmed S.F., Quadeer A.A., Mckay M.R. (2020). Preliminary Identification of Potential Vaccine Targets for the COVID-19 Coronavirus (SARS-CoV-2 ) Based on SARS-CoV Immunological Studies. Viruses.

[83] Lim H.X., Lim J., Jazayeri S.D., Popemma S., Pohl C.L. (2021). Development of multi-epitope peptide-based vaccines against SARS-CoV-2. Biomed. J..

[84] Ihtisham U.H., Kashif R., Muhammad R., Tayyaba A., Sifa A., Kinza Y. (2023). Chapter 18—Polyketides and SARS-CoV-2. Application of Natural Products in SARS-CoV-2.

[85] Ihtisham U.H., Fatima F., Amna S., Abdul B., Firasat H., Israr A., Zarak I.K., Amjad I.A., Faisal S., Umair Y. (2023). Natural Products and SARS-CoV-2. Application of Natural Products in SARS-CoV-2.

[86] Grasselli G., Zangrillo A., Zanella A., Antonelli M., Cabrini L., Castelli A., Cereda D., Coluccello A., Foti G., Fumagalli R. (2020). Baseline Characteristics and Outcomes of 1591 Patients Infected With SARS-CoV-2 Admitted to ICUs of the Lombardy Region, Italy. JAMA.

[87] Wang S.F., Chen K.H., Chen M., Li W.Y., Chen Y.J., Tsao C.H., Yen M.Y., Huang J.C., Chen Y.M. (2011). Human-leukocyte antigen class I Cw 1502 and class II DR 0301 genotypes are associated with resistance to severe acute respiratory syndrome (SARS) infection. Viral. Immunol..

[88] Correale P., Mutti L., Pentimalli F., Baglio G., Saladino R.E., Sileri P., Giordan A. (2020). HLA-B*44 and C*01 prevalence correlates with Covid19 spreading across Italy. Int. J. Mol. Sci..

[89] Li C., Zhao C., Bao J., Tang B., Wang Y., Gu B. (2020). Laboratory diagnosis of coronavirus disease-2019 (COVID-19). Clin. Chim. Acta.

